# Logical model of reception and risk classification for women with pre-eclampsia and eclampsia

**DOI:** 10.1590/1980-220X-REEUSP-2023-0264en

**Published:** 2024-02-02

**Authors:** Sandra Cristina de Souza Borges Silva, Paula Soares Brandão, Gisela Cordeiro Pereira Cardoso, Graciele Oroski Paes, Liana Amorim Correa Trotte, Marluci Andrade Conceição Stipp

**Affiliations:** 1 Universidade do Estado do Rio de Janeiro, Faculdade de Enfermagem, Departamento de Enfermagem Materno-infantil, Rio de Janeiro, RJ, Brazil.; 2 Universidade do Estado do Rio de Janeiro, Faculdade de Enfermagem, Departamento de Enfermagem em Saúde Pública, Rio de Janeiro, RJ, Brazil.; 3 Fundação Oswaldo Cruz, Escola Nacional de Saúde Pública Sergio Arouca, Departamento de Endemia Samuel Pessoa, Rio de Janeiro, RJ, Brazil.; 4 Universidade Federal do Rio de Janeiro, Escola de Enfermagem Anna Nery, Departamento de Enfermagem Fundamental, Rio de Janeiro, RJ, Brazil.; 5 Universidade Federal do Rio de Janeiro, Escola de Enfermagem Anna Nery, Departamento de Metodologia da Enfermagem, Rio de Janeiro, RJ, Brazil.

**Keywords:** Validation Study, User Embracement, Eclampsia, Obstetric Nursing, High Risk Pregnancy, Estudio de Validación, Acogimiento, Eclampsia, Enfermería Obstétrica, Embarazo de Alto Riesgo, Estudo de Validação, Acolhimento, Eclampsia, Enfermagem Obstétrica, Gravidez de Alto Risco

## Abstract

**Objective:**

To describe the validation of the Logical Model of Reception and Risk Classification for women with pre-eclampsia/eclampsia in a high-risk maternity hospital.

**Method:**

Evaluative research with a quantitative approach. The elaboration and validation of the Logical Model were systematized in stages related to the scope review, preparation of the document guided by the Donabedian model and validation by 12 stakeholders, aiming at the assessment of the Content Validation Index.

**Results:**

The problem that gave rise to the intervention was elaborated, supporting the construction of the Logical Model. Agreement was reached on 24 items, reaching a Content Validation Index of 0.99. Stakeholders included contributions regarding correlations between elements of the structure and process.

**Conclusion:**

The document achieved high content validity and could contribute to decision-making by managers in the Reception and Risk Classification sectors for women with pre-eclampsia and/or eclampsia.

## RESUMO

**Objetivo:**

Descrever a validação do Modelo Lógico do Acolhimento e Classificação de Risco às mulheres com pré-eclâmpsia/eclâmpsia em uma maternidade de alto risco.

**Método:**

Pesquisa avaliativa de abordagem quantitativa. A elaboração e validação do Modelo Lógico foram sistematizadas em etapas relacionadas à revisão de escopo, elaboração do documento norteado pelo modelo Donabediano e validação por 12 *stakeholders*, visando à apreciação do Índice de Validação de Conteúdo.

**Resultados:**

Foi elaborado o problema que deu origem à intervenção, subsidiando a construção do Modelo Lógico. Foi alcançada a concordância de 24 itens, alcançando Índice de Validação de Conteúdo de 0,99. Os *stakeholders* incluíram contribuições quanto às correlações entre elementos da estrutura e processo.

**Conclusão:**

O documento alcançou elevada validade de conteúdo e poderá contribuir com a tomada de decisão pelos gestores dos setores de Acolhimento e Classificação de Risco às mulheres com pré-eclâmpsia e/ou eclâmpsia.

## RESUMEN

**Objetivo:**

Describir la validación del Modelo Lógico de Acogida y Clasificación de Riesgo de mujeres con preeclampsia/eclampsia en una maternidad de alto riesgo.

**Método:**

Se trata de una investigación evaluativa de enfoque cuantitativo. La elaboración y la validación del Modelo Lógico se sistematizaron en etapas relacionadas con la revisión de alcance; la preparación del documento se basó en el modelo de Donabedian y fue validada por las 12 partes interesadas, con foco en la apreciación del Índice de Validación del Contenido.

**Resultados:**

Se elaboró el problema que dio origen a la intervención, favoreciendo la construcción del Modelo Lógico. Se llegó a un acuerdo sobre 24 elementos, alcanzándose un Índice de Validación de Contenido de 0,99. Las partes interesadas hicieron aportaciones sobre las correlaciones entre los elementos de la estructura y el proceso.

**Conclusión:**

El documento alcanzó un alto nivel de validez de contenido y podría contribuir a la toma de decisiones por parte de los gestores de los sectores de Acogida y Clasificación de Riesgos de mujeres con preeclampsia y/o eclampsia.

## INTRODUCTION

The manifestations of hypertension during pregnancy are important causes of serious morbidity and maternal mortality in Brazil and around the world. Pre-eclampsia and eclampsia (PE/E) correspond to the main causes of maternal deaths in Latin America and represent the second reason for maternal deaths among Brazilian women. Considering that a large proportion of maternal mortality cases can be avoided with timely care, there is an urgent need for intersectoral actions to strengthen care networks, providing comprehensive care^(1-3)^.

In this sense, *Rede Cegonha* (RC) stands out as a ministerial policy published in 2011, aiming to qualify women’s and children’s health care by guaranteeing continuous and comprehensive care, through articulation between its different components, which develop in different levels of care, namely: Pre-Natal, Childbirth and Birth, Postpartum and Child Care and Logistics System^(4)^.

With regard to care actions in tertiary care, methodologies for classifying care priority, based on listening and clinical assessment of each user, aim to guarantee equity and resolution, as well as favoring access to timely care. The Reception Protocol with Risk Classification (ACCR), a component of the *Rede Cegonha*, indicates that nurses welcome and carry out risk classification in obstetrics according to the degree of urgency, guided by the Manchester System^(4)^.

Reception of the obstetric public presents singularities inherent to the needs and demands related to the pregnancy and puerperal cycle, such as the evaluation of complaints common to pregnancy, which can camouflage clinical situations that require rapid interventions, and also requires the teams involved to use care technologies such as qualified listening and clinical judgment. Thus, the ACCR methodology is a clinical decision support strategy for obstetric emergencies and its purpose is to recognize warning signs of serious or life-threatening cases, providing timely, evidence-based care^(4)^.

The implementation of this care methodology is related to improving the effectiveness and resolution of care for obstetric urgencies and emergencies^(4)^. Although the potential of ACCR is recognized, it is possible to identify some gaps in academic production in searches in bibliographic databases regarding its applicability in the context of high obstetric risk, specifically in the care of women with pre-eclampsia/eclampsia.

The evaluation of a health intervention program allows monitoring of the actions developed, identification of necessary changes and judgment of success in achieving results. To develop an evaluation, it is necessary to understand the structure of the program and correlate the available resources and assistance actions with the expected results^(5,6)^.

The Logical Model (LM) can be defined as a graphical representation of the components of a program, aiming to support decision-making by the management responsible for its improvement. LM can help conceptualize the complexity of the program by describing its components and the relationships among them, explaining the “Theory of Change” and developing hypotheses about the intervention and its possible results. Thus, LM is considered a tool that can guide the development, implementation and evaluation of a program. The construction of LM is made up of different moments, the first is information collection and analysis, which in the case of this study took place through a scope review. The second concerns the pre-assembly of the logical model and can be represented by the problem tree technique, which includes information about the problem, the intervention, general objective, target audience and beneficiaries. The third deals with the validation of the model, which in this case occurred through judgment by stakeholders^(5,6)^.

The participation of professionals interested in the program, also known as stakeholders, in the validation of the LM is essential for different perceptions to be raised about each topic covered, in addition to reaching consensus among those involved. The interaction and agreement between stakeholders represent a factor for LM to operationalize assessments that truly represent the needs of internal and external users of assistance^(5,6)^.

Considering pre-eclampsia/eclampsia as the main cause of maternal morbidity and mortality in the world, and its impact on the well-being of Brazilian women and children, describing the ACCR’s operating theory for this population through an ML will allow analyzing how such actions are developed by their teams in a maternity ward.

It is also noteworthy that the description of the components of the ACCR for women with pre-eclampsia/eclampsia, through modeling, will allow the recognition of its functioning, favoring the production of useful information for care practice and management^(6)^, indicating that Validation of the ML may contribute to supporting strategies for qualifying the care process. The objective of this study was to describe the validation of the Logical Model of Reception and Risk Classification for women with pre-eclampsia/eclampsia in a maternity hospital in the city of Rio de Janeiro.

## METHOD

### Study Design

This is an evaluative research, with a quantitative approach, to evaluate the implementation of reception and risk classification for women with hypertensive syndromes in a maternity hospital. The development of the ML followed the recommendations of the Strengthening the Reporting of Observational Studies in Epidemiology Statement (STROBE)^(7)^, the Institute for Applied Economic Research (IPEA)^(8)^, the Centers for Disease Control and Prevention (CDC)^(9)^ and of the INTEGRATE- HTA Project^(10)^, with some adaptations given the need for social distancing related to the period of the COVID-19 pandemic

### Setting

The study was carried out in a public maternity hospital, a municipal reference in high-risk obstetric care at the tertiary level, located in the central region of the city of Rio de Janeiro.

### Data Collection

**Figure 1:**
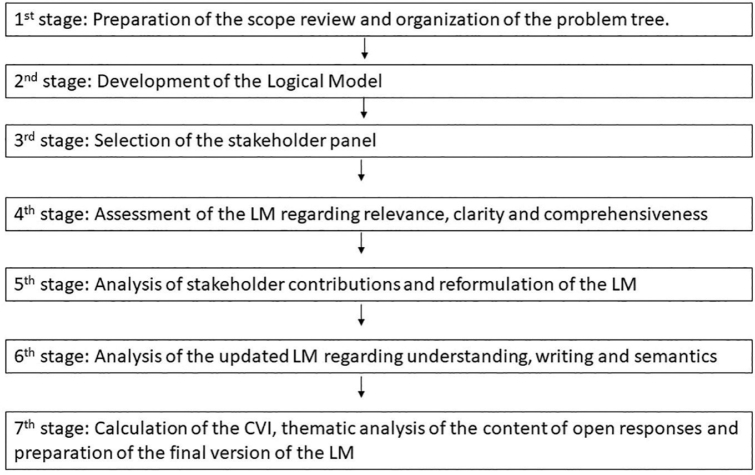
Stages of elaboration and validation of the Logic Model.

The elaboration and validation of the Logical Model took place from October 2021 to April 2022, and took place in 7 stages (Figure 1).

### 1^st^ Stage: Preparation of the Scope Review and Organization of the Problem Tree

The first stage took place from October to December 2021, with a scoping review carried out, following the recommendations of the international guide Preferred Reporting Items for Systematic Reviews and Meta-Analyses for Scoping Reviews (PRISMA-ScR) and the method proposed by Joanna Briggs Institute Reviewer’s Manual 2020^(11)^, based on national and international scientific evidence.

**Figure 2:**
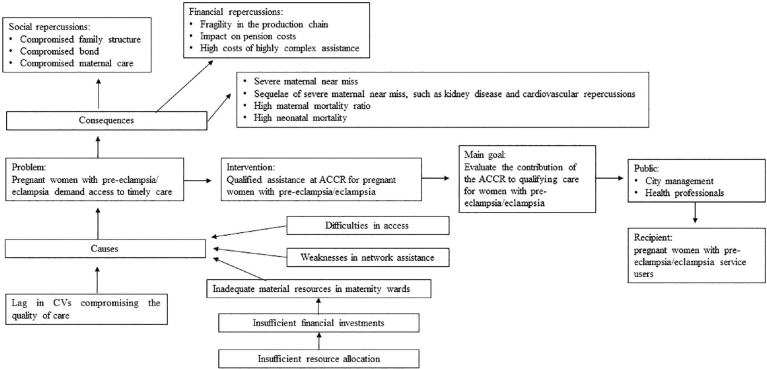
Problems tree.

The searches were carried out in the following databases: Latin American Literature in Health Sciences (LILACS), Nursing Databases (BDENF), Scopus and the Scientific Electronic Library Online (SciELO). The Medical Subject Headings (MeSH) Descriptors “eclampsia”, “pre-eclampsia”, “reception” and “nursing care” were applied in Portuguese and English, resulting in 255 publications, with 14 articles being selected after screening. The findings contextualized with data on morbidity and mortality due to hypertensive syndromes during pregnancy among Brazilian women indicate that qualified assistance actions in Reception and Risk Classification, the gateway to maternity hospitals, can favor access to timely care. Thus, the macro-problem “Pregnant women with pre-eclampsia/eclampsia demand access to timely care” was created, describing its causes and consequences, intervention, objectives, public and beneficiaries, graphically organized in the “problem tree” (Figure 2).

### 2^nd^ Stage: Development of the Logical Model

The information organized in the problem tree supported the elaboration of the Logical Model, based on Avedis Donabedian’s theoretical model, generating a matrix that establishes correlations between the structural resources necessary for assistance, the actions that make up the assistance process and the short and medium results. and long term.

### 3^rd^ Stage: Selection of the Stakeholder Panel

After preparing the LM, the validation process by stakeholders began with the aim of recognizing the instrument’s strengths and weaknesses. To capture validating stakeholders, the “snowball” technique was used, which consisted of locating a key informant group, called “seeds”, which helped the researcher in locating validating stakeholders that fit the selected profile. for research^(12,13)^. These professionals then suggested others using the same selection criteria. This process was repeated until the sampling frame became saturated when reaching a total of 12 participants, in which the same individuals had already been indicated or when no new results were obtained.

Considering that the present study refers to clientele in situations of high obstetric risk, validators who worked in an obstetric care unit with titles of obstetric nurse and master’s degree were considered as inclusion criteria. Those who had worked in the service for less than one year were excluded from the sample.

The sample was initially formed by two key informants selected by the researcher, represented by the nursing technician responsible for the maternity ward of a university hospital and the director of a municipal maternity ward in the city of Rio de Janeiro. Each “seed” indicated two possible participants. Following the “snowball” method, the participants indicated two potential participants, a process repeated until the names of new participants began to be repeated, sample saturation was considered, totaling twelve nurses^(12)^.

It is noteworthy that the researchers formally invited potential participants to collaborate with the research by email. Those who accepted received digital guidance on the objectives and method of the study, and then presented the free and informed consent form (TCLE). This document was signed online by all stakeholders.

### 4^th^ Stage: Assessment of the LM Regarding Relevance, Clarity and Comprehensiveness

At this stage, all stakeholders validated the LM in terms of content and appearance, a judgment that aims to measure the adequacy of the evaluation items in relation to the content, as well as the agreement between the validators. The items’ agreement was assessed in relation to the dimensions of clarity (understanding the text), comprehensiveness (breadth of the phenomenon studied) and relevance (adequacy to the proposed objectives). This process consisted of sending the document to the evaluators through the Google Forms® platform, aiming to obtain a minimum agreement of 80%^(14)^.

The evaluators answered the virtual file composed of clarifications about the validation of the ML and instructions on how to complete it with 5 questions related to the characterization of the professionals and 37 questions that allowed judgment, using a Likert-type scale. At the end of the instrument, there was an open question, which allowed the inclusion of participants’ contributions. A deadline of up to 30 days has been established for returning the instrument.

### 5^th^ Stage: Analysis of Stakeholder Contributions and Reformulation of the LM

The LM analysis by stakeholders supported visual and linguistic reformulations in the document to facilitate connections between items and understanding of the text. The judges advised on enlarging the size of the text boxes to make the content easier to view.

### 6^th^ Stage: Analysis of the Updated LM Regarding Understanding, Writing and Semantics

Subsequently, a virtual file comprising the updated version of the ML and a form with eight open questions was sent to stakeholders to evaluate the document in terms of understanding, writing and semantics. A 30-day deadline was agreed for sending responses to researchers.

### 7^th^ Stage: Calculation of the CVI, Thematic Analysis of the Content of Open Responses and Preparation of the Final Version of the LM

Analysis of the results allowed the preparation of the final version of the ML and the determination of the Content Validation Index (CVI). The quantitative data results were saved in Microsoft Excel® 2010 spreadsheets and analyzed using descriptive statistics using the Excel program (Microsoft, USA). The CVI was calculated by adding the criteria presented and dividing by the total number of responses. Items that reached more than 80% agreement between judges and CVI >0.80 were considered valid, so items that presented indexes below the minimum were reformulated according to the judges’ assessment (9.12).

Recent studies on the development of measuring instruments demonstrate that a widely used approach to assess content and/or appearance validity is the CVI per item, per dimension or overall, adopting a minimum value to achieve agreement, which can vary from 0.70 to 0.80^(13,14)^.

### Ethical Aspects

The research was approved by the Research Ethics Committees of the Anna Nery School of Nursing at the Federal University of Rio de Janeiro and the Municipal Health Department of Rio de Janeiro, as it met the ethical and legal guidelines of Resolution No. 466/2012 of the Council. National Health Authority, which regulates and guides the development of research involving human beings. Due to the pandemic context, Free and Informed Consent was obtained from all individuals involved in the study by signing the Free and Informed Consent Form in the online format.

## RESULTS

The group of stakeholders was characterized by women, with an average age of 42 years, graduated 15.2 years ago. The participants had an average time working in obstetric care of 13.5 years. Of the twelve judges, 8 worked in direct care in an obstetric unit. In the first round of validation, agreement was observed in most of the items described in the document, thus, the CVI reached 0.92 (Chart 1). Of the 24 items evaluated by the experts, two did not reach the minimum CVI, with the topics “Ensuring the presence of a companion of the woman’s choice” and “Quantity of women with a companion attended” being excluded. The item “Referral of stable women who need to return to prenatal care” was re-presented in the form “Referral of stable women to prenatal care”, following the suggestion of the appreciators.

**Chart 1:** Content validation of the LM elements of assistance actions for women with pre-eclampsia/eclampsia in the ACCR in a high-risk maternity hospital – Rio de Janeiro, RJ, Brazil, 2023.

Component	Analyzed Elements	CVI
**Structure**	Evidence-based standards, techniques and protocols	1
	Professional qualification	1
	Ambience	0,8
	Systematized regulation system	1
	Systematized removal system	1
**Process**	Diagnosis and assistance for women with pre-eclampsia/eclampsia	1
	Risk rating	0,8
	Guarantee of the presence of the woman’s chosen companion	1
	Identification and treatment of severe forms of pre-eclampsia/eclampsia	0,8
	Assessment of fetal well-being	1
	Referral of stable women who need to return to prenatal care	0,8
	Referral of unstable women who require hospitalization	1
	Removal of women who need to go through regulation	1
**Short-term result**	Number of services and risk classifications	0,8
	Number of women with companions served	1
	Number of women referred	0,8
	Number of stable women referred to prenatal care	0,8
	Number of women diagnosed and treated early	1
**Medium-term result**	Reducing the rate of neonatal asphyxia	0,8
	Reduction in maternal near miss ratio	0,8
	Reduction in the frequency of maternal, fetal and neonatal complications due to pre-eclampsia/eclampsia	1
**Long term result**	Improving the quality of intervention	1
	Reduction in the maternal mortality ratio in the institution	0,8
	Reduction in early and late neonatal mortality rates in the maternity ward	1

In the second round, changes were suggested in the visual and linguistic presentation of some items, facilitating connections between items and understanding the text. Stakeholders recommended increasing the size of the text boxes and using colors on the arrows to facilitate correlation between content. The items “Trained Professionals” and “Early Diagnosis and Treatment Performed” were suggested to be replaced by the sentences “Trained Nursing Professionals” and “Medical Diagnosis and Early Treatment Carried Out”, highlighting the professional category involved in the procedural action.

The participants did not broadly agree that “professional qualification favors stable women being referred to prenatal care, according to the identified need” and “The structured regulatory system favors the referral of unstable women who require hospitalization”. Thus, stakeholders correlated success in network actions with access to timely care.

The experts reported that the effectiveness of the ACCR is related to the components of the physical structure of reception in the items: “The ambiance of the ACCR, when guided by RDC 36/2008, favors the effectiveness of assistance to women with pre-eclampsia/eclampsia”, “The The ambience of the ACCR, when guided by RDC 36/2008, favors the identification and treatment of manifestations of severe pre-eclampsia and eclampsia” and “Permanent and disposable devices necessary for care favor the effectiveness of care for women with pre-eclampsia/eclampsia”.

**Figure 3:**
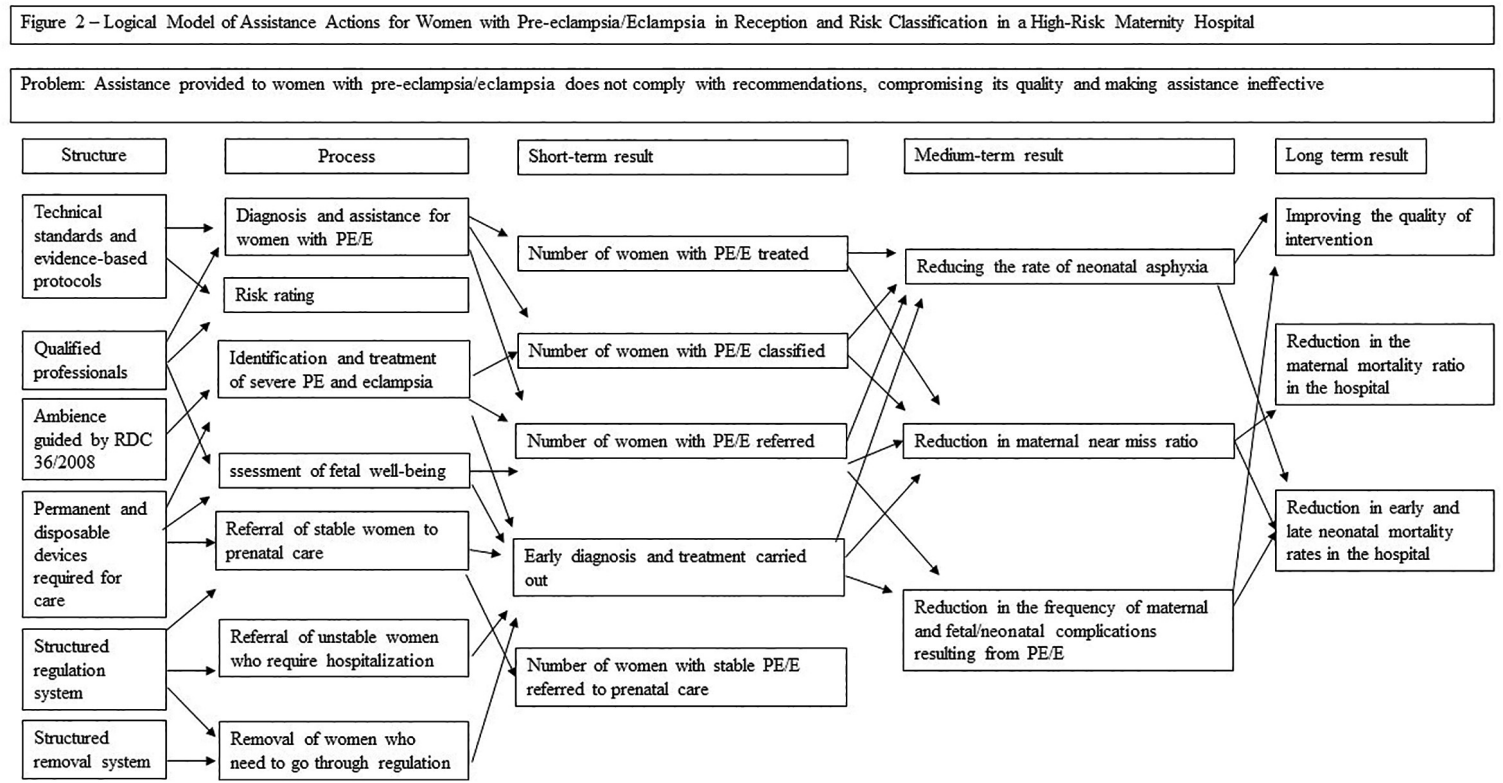
LM of care actions for women with pre-eclampsia/eclampsia at ACCR in a high-risk maternity hospital.

The suggested changes were evaluated for relevance by the researchers and included, generating the LM version (Figure 3).

## DISCUSSION

LM evaluators are characterized as women in the fourth decade of life, having worked in obstetric care for a period exceeding 80% of their training time, with a higher prevalence of insertion in direct care. It is necessary to consider that sociodemographic and training characteristics have a direct relationship with the monitoring of transformations in society, as well as the insertion of advances in soft, soft and hard technologies in the care field, requiring the development of strategies that promote meeting needs system users and improving team working conditions^(15)^.

The results found indicate that the methodology proposed by the study provided a consensus among stakeholders in validating the logical model. The percentage of agreement achieved was greater than 0.80, the minimum established in other validation studies^(12,13)^. In the scope of obstetric care, studies were developed that modeled interventions, with the purpose of recognizing the aspects that should be evaluated, understanding that the schematic representation of the ML prints the program’s operating theory so that the defined objectives are achieved^(9,12,14)^.

For the effectiveness of assistance at ACCR, the ambience in accordance with ministerial guidelines is as important as the communication skills that make up the reception practice. The appreciation of humanizing attitudes related to communication skills (qualified listening to complaints, fears and expectations and treatment of users by their own name) by experts corroborates the conceptual basis of welcoming, a care technology of a care model that seeks to overcome verticality of actions between professionals and users^(15)^.

The practice of welcoming is related to active and qualified listening, transversal to the act of caring, whose potential can be affected by weaknesses in the work process, such as long working hours, inadequate staffing related to the pandemic context^(16,17)^ and overcrowding at the entrance door related to the mistaken search for care in a tertiary unit due to weak links with the basic network^(16,17)^.

Women during the pregnancy and puerperal cycle often associate the way they are treated by professionals with maternity care. Thus, they attribute the ability to communicate and individual characteristics such as patience, attention and cordiality as determinants of the way in which assistance is established. Therefore, human relationships are a decisive aspect in determining the bond and trust between professionals and users of the health service^(17,18)^.

The presence of a companion, a right guaranteed by Federal Law 11,108/2005, addresses another aspect related to humanizing attitudes^(18-20)^. The experts did not establish a direct relationship between the emotional support offered by the companion and the effectiveness of the care process for women, since pre-eclampsia and eclampsia are diseases that require diagnostic and therapeutic actions within the scope of high complexity, with this being excluded. LM item at the suggestion of the participants.

There is evidence that the presence of a companion, in addition to providing emotional support to women, is a marker of safety and quality of care by avoiding violence and inappropriate birth practices^(19-21)^. However, no studies were found that relate its presence and care practices at ACCR to hypertensive patients.

The experts conditioned the effectiveness of assistance at ACCR to timely care at different levels of care, through the vacancy regulation system. Weaknesses in reception and the difficulty in communicating between services in providing timely care result in the maintenance of a significant percentage of pilgrimages seeking assistance. Such factors are related to the degree of implementation not suitable for obstetrics reception in the ACCR identified in the *Rede Cegonha* evaluation. Strengthening the comprehensiveness of network care is related to the appropriate targeting of resources, efficiency of the health system and non-fragmentation of care^(20-22)^.

## CONCLUSION

The construction of the LM and its validation by experts showed that the physical structure of hospital environments and reception as components of the ACCR surpass physical plants and functional aspects. They imply enhancing humanizing practices in the interaction between professionals and the women they serve, which can produce multidimensional and health-promoting care. Thus, the ACCR aspects raised produced a “web” crossing multiple “threads”, subjects and factors: families, women, equity, comprehensiveness, care coordination, acceptability and opportunity.

The experts suggested the equivalence in the relevance of soft and hard technologies for comprehensiveness and timely care when they pointed out that the effectiveness of assistance, in this context, mutually depends on welcoming and ensuring access to different levels of care.

The study has limitations as it presents results related to a high obstetric risk unit, suggesting the need for replication in other units with the same characteristics. However, the results contribute to management by relating the need for actions that enable the connection of pregnant women with health units, a structure compatible with care needs, avoiding users’ pilgrimages.

Another contribution refers to the field of professional training, in which it is necessary to enhance actions that favor the development of skills and competencies for qualified listening and the capacity for precise clinical judgment, recognizing the specificities in care in the pregnancy and puerperal cycle.
